# The Effect of Auricular Massage on Naso-Oral Suctioning Procedural Pain in Premature Neonates: A Randomized Controlled Crossover Study

**DOI:** 10.1155/tswj/8819743

**Published:** 2025-10-07

**Authors:** Reem A. Ali, Roa'a F. Obeidat, Arwa I. Oweis

**Affiliations:** Department of Maternal and Child Health Nursing, Faculty of Nursing-Jordan University of Science and Technology, Irbid, Jordan

**Keywords:** auricular therapy, infants, Jordan, massage, nasopharyngeal suctioning, NICU, pain management, premature

## Abstract

**Background:**

In the neonatal intensive care unit (NICU), premature neonates frequently endure painful procedures that can lead to long-lasting sequelae, underscoring the critical need for safe and effective pain management strategies. Auricular massage has demonstrated significant benefits for children, including anxiety and stress relief. Investigating its effectiveness in alleviating pain in neonates could introduce a valuable nonpharmacological approach to pain management, enhancing the overall care and comfort of these vulnerable infants.

**Objective:**

This research is aimed at studying the effectiveness of a 3-min session of auricular massage in reducing pain caused by nasal and oral suctioning among premature neonates in the NICU in Jordan.

**Methods:**

A randomized controlled trial employing a single-blind crossover design was conducted in a single hospital. Preterm neonates were randomly assigned to one of two conditions. In Condition I, neonates initially received auricular massage treatment after suctioning, followed, after 2 days, by no massage after suctioning. Conversely, in Condition II, neonates had the reversed sequence, starting with no massage and then receiving auricular massage treatment after 2 days. Pain levels in the neonates were assessed using the Premature Infant Pain Profile-Revised (PIPP-R) scale.

**Results:**

Data from 60 preterm neonates were analyzed using the SPSS Version 28. Chi-square and independent *t*-tests revealed no significant differences between neonates in the study conditions based on gender (*p* = 0.43), gestational age (*p* = 0.41), and body weight (*p* = 0.35). Paired *t*-test results indicated a significant difference in pain scores when comparing periods of auricular massage to periods without massage. The mean pain score following auricular massage was 3.63 (SD = 2.36), whereas it was 10.23 (SD = 2.40) in the absence of massage.

**Conclusion:**

Auricular massage for a 3-min duration is an effective nursing intervention that warrants consideration as a nonpharmacological method of pain relief for premature neonates during admission to the NICU.

## 1. Introduction

Preterm birth is a significant global health issue, with approximately 13.4 million infants born prematurely each year, representing 4%–16% of all live births in 2020 [1]. The prevalence of preterm birth is notably high in developing and underdeveloped countries [1]. Jordan, as a developing country, experiences a high prevalence of preterm births, with a reported rate of 8.7 per 100 live births in 2020 [2].

Advances in medical technology have significantly improved the survival rates of premature neonates. Many of these neonates are placed in neonatal intensive care units (NICUs) for therapeutic management, where they are exposed to numerous stressful and painful stimuli [3]. According to a systematic review, neonates in the NICU undergo an average of 7.5–17.3 painful procedures per day [4]. Most premature neonates experience respiratory challenges necessitating ventilation support [3, 5], which further exposes them to painful procedures [3]. Respiratory support and associated procedures, such as endotracheal intubation, naso-oral, and pharyngeal suctioning, cause significant pain and stress for neonates [5–7].

Premature infants have immature pain pathways, rendering their ability to modulate painful stimuli less effective [8]. Consequently, they not only experience pain but respond to it more severely compared to term neonates [9, 10]. Pain has both short- and long-term consequences on preterm neonates' health and well-being [11], including accelerated heart rate (HR) and decreased oxygen (O_2_) saturation levels [12]. Pain is also detrimental to neonatal brain development [13–15], potentially leading to maladaptive behavior and future learning problems [16]. Therefore, effective pain management for neonates in the NICU is a prime concern for healthcare professionals. Pharmacological methods, such as opiates, used to manage severe pain are not without risks; they can cause respiratory depression, physiological alterations, and seizures [17]. Additionally, administering opiates is sometimes contraindicated for neonates on ventilation support [18]. For alleviating less severe pain, nonpharmacological methods can be beneficial [19].

Nonpharmacological pain management has been found to be effective and well-tolerated by neonates [10, 20]. Various nonpharmacological methods for pain management are used for neonates, including kangaroo care, nonnutritive sucking (with and without sucrose), swaddling, tucking, breastfeeding, acupuncture, acupressure, body touch/massage, and auriculotherapy [21–27].

Evidence indicates that premature neonates can benefit from nonpharmacological pain relief methods such as body touch/massage and auricular acupressure [21, 22, 28–34]. Auriculotherapy traditionally involves the stimulation of specific points in the ears that correspond to various parts of the body, based on body morphology [35]. According to the battlefield concept of auriculotherapy, there are five key points in the ears believed to modulate pain in the central nervous system: the cingulate gyrus, the thalamus point, Omega 2, point zero, and Shenmen [36]. Therapeutic stimulation of these points can be achieved using needles, laser, or pressure [36].

Evidence on the suitability of auricular interventions as a pain relief method for premature neonates is limited. Only two studies have examined the effects of noninvasive magnetic acupuncture on procedural pain in premature infants [21, 22]. No studies have been located specifically focused on the benefits of auricular massage as a pain relief method for neonates in the NICU. Additionally, there is no standardized auricular massage protocol for premature infants available in the literature. This highlights the need for further research to establish evidence-based guidelines and protocols for auricular massage in this vulnerable population.

Emerging evidence on the analgesic effects of auricular massage in children [37, 38], along with promising findings on auricular acupressure for pain relief in premature infants, has prompted further inquiry into its potential efficacy within neonatal intensive care settings. Auricular massage—encompassing gentle techniques such as light rubbing, friction, and acupressure applied manually to the external ear—may offer meaningful relief from pain in neonates. This approach is particularly feasible for busy neonatal nurses working in the NICU. It requires minimal training, involves no specialized equipment, and can be performed using the hands while the infant remains inside the incubator [39]. The procedure is brief, typically lasting only a few minutes, and is unlikely to overstimulate premature infants, making it a practical and low-risk adjunct to routine care.

Neonatal nurses are pivotal in the management and alleviation of pain in premature neonates. Their responsibilities encompass assessing pain levels, implementing pain management strategies, and ensuring the comfort and well-being of neonates [40] during painful procedures such as naso-oral suctioning. Given the critical role of neonatal nurses in pain management, we hypothesize that the pain scores induced by naso-oral suctioning in preterm neonates will be significantly reduced following auricular massage treatment compared to the pain scores observed during periods without auricular massage intervention.

Although suctioning is one of the most frequently performed painful procedures in the NICU [7, 41], the majority of existing research has primarily focused on procedural pain related to heel pricks, blood sampling, and umbilical catheterization [21, 25–27]. Notably, only a limited number of studies have explored the analgesic effects of nonpharmacological interventions—such as gentle human touch—during endotracheal and airway suctioning [31, 33].

However, based on the research team's comprehensive review, no studies to date have specifically investigated the efficacy of auricular massage in alleviating naso-oral suctioning-induced pain in premature infants, either within Jordan or globally. Therefore, the present study is aimed at evaluating whether auricular massage can serve as an effective nonpharmacological strategy for mitigating suction-related pain in premature neonates admitted to NICUs in Jordan.

## 2. Methods

### 2.1. The Aim

The aim of the current study was to investigate the effect of auricular massage on pain response after naso-oral suctioning among premature neonates who were on noninvasive ventilation support in the NICU in Jordan.

### 2.2. Design and Sample Size

A randomized controlled crossover interventional study was conducted from January to September 2023. The study took place in a NICU at a Level III hospital affiliated with a university. Approval was obtained from both the university and the hospital's Institutional Review Boards (IRBs). For the purpose of this study, all eligible neonates in the selected NICU were randomly assigned to two conditions following parental consent. In Condition I, neonates initially received auricular massage treatment after suctioning, followed, after 2 days, by no massage after suctioning. Conversely, in Condition II, neonates had the reversed treatment sequence, starting with no massage after suctioning and then, after 2 days, receiving auricular massage treatment after suctioning. Although crossover designs are traditionally employed to compare multiple interventions, it is well documented in the literature that one of the conditions may involve the absence of intervention [31, 37, 42–44]. This approach is particularly appropriate when the primary objective is to control for within-subject variability and enhance statistical power. In the context of studying premature infants in the NICU—a population characterized by clinical vulnerability and ethical constraints—a crossover design offers a methodologically sound and ethically favorable framework. It allows for maximizing data obtained per infant while minimizing overall sample size, thereby increasing the sensitivity to detect the effects of auricular massage as a nonpharmacological intervention for pain relief.

The required sample size for a 2 × 2 crossover design with an effect size of 0.5, a significant level of 0.05, and a power of 80% is 62.72 infants. Taking into consideration a potential 15% attrition rate, the target sample size was around 72 premature infants.

### 2.3. Participants

Neonates were enrolled in the study based on specific inclusion criteria. Inclusion criteria required that the neonate's gestational age be between 28 and < 37 weeks, with an Apgar score of 7 or higher at the fifth minute of birth, and a current weight of ≥ 1000 g. Additionally, the neonate needed to be on noninvasive ventilation support, such as continuous positive airway pressure (CPAP) or Vapotherm, and require regular suctioning every 4–6 h. Neonates should not have received any opiates or sedatives within the 4 h prior to the study intervention and should not have undergone any other painful procedures in the hour preceding the study intervention. Also, neonates should not have major medical illnesses, congenital malformations, or intracranial hemorrhage Grade III or IV, or who were medically unstable. In the NICU where the study was conducted, the use of other nonpharmacological procedures for pain relief was not standardized. Therefore, no omission of care resulted as a consequence of the study.

### 2.4. Measures

#### 2.4.1. Demographic Characteristics

Neonatal characteristics were systematically collected using an abstraction sheet developed by the researchers, grounded in existing evidence. The collected characteristics included gestational age, mode of birth, current weight, gender, and Apgar scores at birth.

#### 2.4.2. Neonatal Pain

The dependent variable in this study is neonatal pain following the study treatments (auricular massage/no massage), measured using the Premature Infant Pain Profile Revised (PIPP-R). The PIPP-R is a comprehensive pain assessment tool that incorporates seven indicators: physiological factors: changes in HR and reduction of O_2_ saturation; contextual factors: gestational age and behavioral state; and behavioral indicators: facial reactions including brow bulge, eye squeeze, and nasolabial furrow [45]. These indicators are rated on a 4-point Likert scale (0–3). The total PIPP-R score, ranging from 0 to 21, is calculated based on these seven indicators. Higher scores indicate greater levels of neonatal pain, with scores of 1–6 indicating *mild pain*, 7–12 indicating *moderate pain*, and scores above 12 indicating *severe pain* [45]. The PIPP-R is highly valid and reliable for measuring pain in premature infants, provided that users receive adequate training [45, 46]. Given that the PIPP-R integrates both physiological and behavioral indicators, interrater reliability was assessed in the current study. The interrater correlation coefficient between two observers (trained nurse who assessed neonatal pain and one trained researcher) was 0.92, indicating excellent agreement.

### 2.5. Auricular Massage

Due to the absence of a standardized auricular massage protocol for premature infants, the auricular massage intervention was developed by the researchers based on previous studies on infant massage techniques [25, 27, 29, 30, 43, 47, 48], guided by the International Association of Infant Massage guidelines [49], and studies on auricular acupuncture and acupressure for children [21, 22, 36, 37, 50–54].

**Figure 1:**
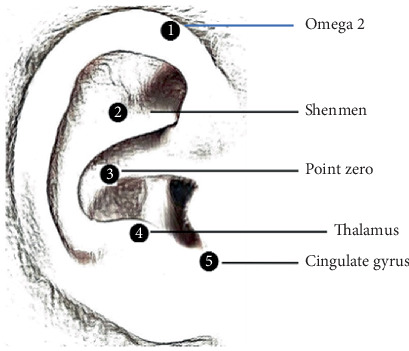
Battlefield acupressure points.

The auricular massage protocol consists of six steps, lasting 3 min. First, the researcher holds the neonate's ears with warm fingers for 60 s to provide light human touch. The second step involves using the index and middle fingers in a V shape to provide gentle, circular massage of the ears, moving from down to up for 15 s (a total of seven turns). This step is based on the “battlefield acupuncture” concept presented by Niemtzow [36], where stimulation of five auricular acupoints can attenuate pain in minutes. These five auricular acupoints are shown in Figure 1. Due to the small size of premature neonates' ears, identifying the exact location of the auricular acupoints—cingulate gyrus, point zero, Shenmen, thalamus, and Omega 2—proved challenging, so the front and back of the ear were considered acupoint zones. Circular massaging the ears from the front and back stimulates four of the battlefield acupoints (cingulate gyrus, Shenmen, thalamus, and Omega 2). The third step involves performing a circular motion massage by the index finger on the skin in front of and behind the ears for 15 s (a total of three turns) to stimulate blood circulation. The fourth step, also based on the battlefield technique, involves using the index finger and thumb to apply gentle pressure on the folds of the ears, starting from the root of the helix and covering the triangular fossa, antihelix, scapha, antitragus, and moving toward the ear lobe for 15 s (a total of three turns). The fifth step involves providing gentle pressure and friction on the ear lobes for 15 s. The ear massage concludes with another episode of the researcher holding the ears between her fingers for 60 s. This innovative auricular intervention combines human touch, massage, and acupressure to comfort neonates after suction.

The face validity and safety of the auricular massage protocol were reviewed and approved by a professor specializing in developmental care approaches and clinical neonatal nurses before obtaining approval from the university and hospital's IRBs. The researcher received training on the use of the developed protocol and subsequently practiced intensely on older children before performing the massage on neonates to master the steps while ensuring safety. Upon parental approval, the developed auricular massage was piloted on six neonates according to the study design. During the implementation of the auricular massage, the neonate was placed in a supine position while the researcher faced the neonate. During the massage, the incubator was closed, and the nurse performed the massage through the incubator's windows to avoid altering the temperature. All neonates in the pilot study were carefully observed for any possible consequences of the auricular massage. None of the neonates in the study experienced any adverse effects due to the auricular massage.

### 2.6. Ethical Considerations

Ethical approval for the study was obtained from the IRBs of both the university and the hospital (Approval #2022/585). Informed consent was secured from the parents of premature neonates after providing them with a comprehensive explanation of the study's purpose, protocol, potential risks, and benefits. Open communication with parents was maintained throughout the study, ensuring they were aware that they could withdraw their neonates from the study at any time without any negative consequences. The neonates were closely monitored for any adverse effects during the study period.

### 2.7. Study Procedure

**Figure 2:**
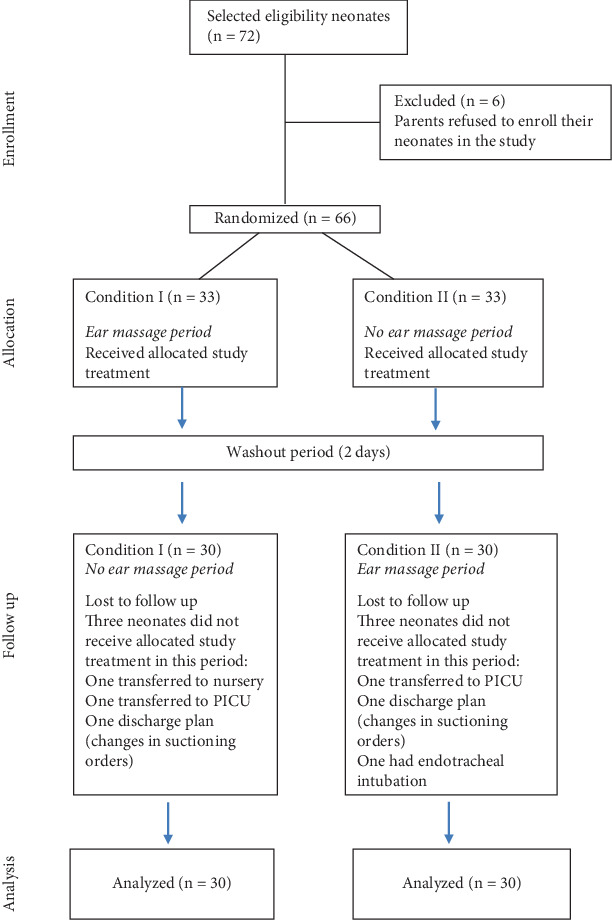
Diagram illustrating the progression of premature neonates through study conditions in each period of a randomized trial.

Upon obtaining the ethical approval to conduct the study, 72 neonates were selected based on the study criteria from the NICU across the study period. Of all the parents of neonates who were approached, only six declined to participate in the study. They refused participation due to disinterest or concerns about potential discomfort to their neonates. Neonatal demographic data were collected from medical records and nursing notes using the abstraction sheet. In this crossover study, neonates were randomly assigned via a coin flip to one of two treatment sequences (conditions). For neonates assigned to Condition I (heads), 33 participants first received a 3-min auricular massage immediately after suctioning, with their pain levels assessed immediately following the intervention. Two days later, these same neonates underwent suctioning without receiving any massage, and their pain levels were measured 3 min postsuctioning. In contrast, neonates assigned to Condition II (tails) received the treatments in the reverse order: they initially underwent suctioning without the massage, with pain assessed 3 min after the procedure, and then, 2 days later, they received the 3-min auricular massage immediately after suctioning with a subsequent immediate pain assessment. This reversed treatment order ensured that every neonate acted as their own control, eliminating individual variability and allowing a direct comparison of the effect of auricular massage versus no massage. As illustrated in Figure 2, six neonates were excluded from the study, resulting in a final sample size of 60 neonates.

To ensure consistency throughout the study, suctioning and pain assessments for all neonates were conducted by one nurse, who was blinded to the neonates' condition assignments. This nurse held a master's degree and had over 5 years of experience working in the NICU. Immediately after performing suctioning according to NICU protocol, the nurse recorded the neonate's baseline HR (highest reading) and O_2_ saturation (lowest reading) from the attached monitor, following the guidelines of the PIPP-R scale. The nurse then left the neonate with the researcher, who either performed a 3-min auricular massage (auricular massage period) or observed the neonate for 3 min (no auricular massage period), depending on the neonate's condition assignment. Upon completion of the study treatment, the researcher signaled the nurse to return and complete the pain assessment using the PIPP-R scale. The washout period of 2 days was determined based on prior studies that employed nonpharmacological methods for pain management in preterm infants [43, 44, 55]. A washout period was implemented to minimize potential carryover effects and reduce bias related to the order of intervention administration.

The nurse responsible for performing suctioning and pain assessments for all neonates received comprehensive training on using the PIPP-R scale from the principal researcher. Practice sessions were conducted to ensure a high correlation between the nurse's and the researcher's pain scores using the PIPP-R. Additionally, auricular massage was administered to all neonates by a single researcher who was thoroughly trained by the professors who developed the massage protocol.

### 2.8. Data Analysis

Statistical analysis was done by SPSS Version 28 for Windows (SPSS Inc., Chicago, IL, United States). Percentages and frequencies were used to provide a general description of the study sample. Means and standard deviations for study groups were compared using independent and paired *t*-tests and chi-square tests. The Wilcoxon signed-rank test was employed to analyze PIPP-R subscale scores, given the ordinal nature of the grading system: 0 = *no pain*, 1 = *minimal pain*, 2 = *moderate pain*, and 3 = *maximum pain*. The alpha significance level was considered at 0.05.

## 3. Results

### 3.1. Sample Demographic Characteristics

The demographic characteristics of the 60 neonates in the study are shown in Table 1. The number of male neonates (58.3%) is slightly higher than that of female neonates, with a mean gestational age of 34.3 weeks (SD = 1.78). The majority (70%) of neonates were singletons and born by cesarean section. The average current weight for neonates was 1860 g (SD = 530), and 80% of them had an Apgar score of 8–9 points at 5 min after birth. There were no significant differences between neonates in both conditions (I and II) with respect to gestational age, gender, and current body weight (Table 2).

### 3.2. Neonatal Pain Scores

Mean neonatal pain scores, as measured by the PIPP-R, for the two conditions are presented in Table 3. The results indicated that during periods of auricular massage treatment, the mean neonatal pain scores for Condition I and Condition II were 3.77 and 3.50, respectively, with no significant difference between conditions, as evidenced by independent *t*-test results (*p* = 0.67). Additionally, during periods without massage treatment, the mean pain scores for neonates in Condition I and Condition II were 10.47 and 10.00, respectively, with no significant difference between conditions (*p* = 0.46) which indicates no carryover effect of the auricular massage.

### 3.3. Effectiveness of Auricular Massage

The study results indicated that 75% of neonates experienced moderate pain and 16.7% experienced severe pain 3 min after suctioning in the absence of auricular massage. In contrast, only 13.3% of neonates experienced moderate pain, and none experienced severe pain following the auricular massage period. Chi-square statistics revealed a significant difference in pain levels between the study periods (massage/no massage); *χ*^2^ = 74.58, *p* < 0.0001 (Table 4).

The number needed to treat (NNT) was calculated for the events of pain reduction (proportion of infants experiencing mild pain) during the massage periods compared to the absence of massage periods. The absolute risk reduction (ARR) was 0.7833 (78.33%), and the NNT to achieve one additional case of pain reduction (mild pain) with ear massage compared to the absence of ear massage was 1.28 infants, further supporting the effectiveness of ear massage in reducing pain in infants.

When comparing the mean neonatal pain scores using a paired *t*-test, there was a significant difference in pain scores between the periods when premature neonates received auricular massage (M = 3.63, SD = 2.36) and when they did not receive massage (M = 10.23, SD = 2.40). A significant decline in the mean pain scores (M = 6.60, SD = 2.25) for neonates after auricular massage was evident; *t* (59) = 22.73, *p* < 0.0001. The effect size, calculated using Cohen's *d*, was 2.94, indicating that the average pain levels for premature neonates during auricular massage were approximately three standard deviations lower than the average pain levels during periods without massage. This large effect size further underscores the practical significance of auricular massage in alleviating pain for premature neonates. Furthermore, the results of the PIPP-R subscale analysis (Table 5) demonstrated a significant reduction in neonatal pain scores across all subscales during the period when auricular massage was administered following suctioning compared to the period without massage.

## 4. Discussion

The current investigation provides insight into neonatal pain associated with naso-oral suctioning and evaluates auricular massage as a nonpharmacological intervention for pain relief—an area that remains underexplored in the existing literature. Findings of our study align with existing studies on auricular-related interventions in premature infants, demonstrating a clear consensus. Previous research has established a positive correlation between auricular interventions, such as noninvasive magnetic acupuncture, and pain relief during procedures like eye exams for retinopathy [22] and heel pricks [21]. In these studies, magnetic stickers were placed on the infants' ears at acupuncture points according to the battlefield protocol to stimulate these points for pain relief. In our study, we applied light pressure on auricular acupoints to reduce pain. Additionally, our findings showed no adverse effects, such as skin irritations or discomfort, consistent with other evidence from auricular intervention studies [21, 22]. Due to the limited availability of studies specifically investigating the efficacy of auricular massage for pain management in premature neonates, comparative interpretations in the current study were informed by existing research on ear-related interventions, body massage, and gentle human touch—each recognized as nonpharmacological strategies for pain relief among premature neonates.

The study findings reveal that naso-oral suction is a moderately painful procedure as evident by PIPP-R mean scores of 10.47 and 10.0 during periods of the absence of auricular massage; this finding is consistent with previous studies. Using the same pain scale, Oliveira et al. [33] and Fatollahzade et al. [31] reported that during suctioning, premature neonates showed mean pain scores of 10.0 and 8.97, respectively. As well, Luo et al. [7] reported that nasopharyngeal suctioning is a painful procedure to premature neonates as measured by the Neonatal Infant Pain Scale.

The current study demonstrated statistically significant differences in total pain scores between periods when neonates received auricular massage and when they did not. These findings align with those of Fatollahzade et al. [31], who conducted a randomized controlled crossover trial assessing the analgesic effect of gentle human touch in 34 premature neonates during endotracheal suctioning in the NICU. Notably, the reduction in pain scores observed in our study following auricular massage was more pronounced (*t* = 22.73) compared to the reduction reported by Fatollahzade et al. (*t* = 3.4). Furthermore, the frequencies of moderate (64.7%) and severe (2.9%) pain levels in their study were substantially higher than those recorded after auricular massage in our cohort, where only 13.3% of neonates experienced moderate pain and none exhibited severe pain. This difference may be attributed to the dynamic nature of auricular massage, which involves friction and circular motion—mechanisms that may stimulate endorphin release [56]. Additionally, targeted pressure on battlefield acupuncture points within the auricle may enhance pain modulation through central nervous system pathways. This mechanism is supported by Liu et al. [57], whose systematic review concluded that auricular acupressure exerts a significant analgesic effect and is effective in managing various types of pain.

In the current study, behavioral indicators of pain—specifically brow bulge, eye squeeze, and nasolabial furrow—were significantly reduced following auricular massage in premature neonates. These findings are consistent with those reported in a crossover randomized trial involving 50 premature neonates who received gentle human touch during suctioning [33]. However, our study additionally demonstrated greater physiological and behavioral stability, as evidenced by minimal changes in HR, less reduction in O_2_ saturation, and maintenance of an active–quiet awake state following auricular massage. In contrast, Oliveira et al. did not observe such autonomic improvements, which may be attributed to differences in assessment timing. In Oliveira et al.'s study, pain was evaluated during the suctioning procedure itself, whereas in our study, pain was assessed following auricular massage, which likely allowed for enhanced neonatal autonomic regulation and recovery. This explanation is supported by existing literature indicating that massage interventions can enhance O_2_ saturation and stabilize HR in premature neonates [58, 59].

Findings of this study suggest that auricular massage offers significant benefits for neonates in clinical practice. As the first investigation into the impact of auricular massage on pain caused by suction in premature neonates, replication of this study is essential to further validate or challenge our findings. Nonetheless, the robustness of our study is supported by several key aspects. Utilizing a crossover design, premature neonates served as their own control, thereby minimizing variability in pain scores due to confounding factors. The suctioning procedure was consistently performed by a single nurse according to hospital protocol, ensuring uniformity across the study. Similarly, the auricular massage intervention was administered by one researcher for all neonates. Additionally, the study employed a well-established pain assessment scale specifically designed for premature neonates. The nurse assessing pain levels was blinded to the neonate's condition assignment, further enhancing the study's rigor.

## 5. Implications

Nonpharmacological pain-relief measures, such as auricular massage, warrant high consideration by NICU nurses, as indicated by the present study's results. Auricular massage was found to be accessible for premature neonates reliant on ventilation devices such as Vapotherm and CPAP. This intervention requires no equipment to apply and has no evident adverse effects, such as skin irritation or signs of discomfort. Neonatal nurses can benefit from this study by adopting auricular massage at no cost as part of nursing care, not only to comfort neonates after suction but also to provide more human touch in a high-tech environment. This approach allows nurses to become more familiar with each neonate's status and behaviors while providing developmental care that aligns with the neonate's sense of touch, the most developed sense in newborns [60]. Furthermore, introducing auricular massage as a nonpharmaceutical pain relief method could reduce the reliance on pharmaceutical methods, which require more attentive care and incur higher costs in the NICU.

Nurses can train parents to perform auricular massage for their neonates whenever it is feasible. Substantial evidence supports the benefits of parental involvement in the care of neonates in the NICU, for both the neonates and the parents. For instance, Geary et al. [61] conducted a narrative review of 323 studies examining the effects of mother-led infant massage. The review revealed that maternal involvement in infant care reduced symptoms of postnatal depression and improved the mother–infant relationship, in addition to providing benefits for the infants.

Currently, there are no clinical guidelines for implementing auricular massage for premature neonates in Jordan, and this practice appears unstandardized globally. However, adopting the current study protocol could serve as a foundation for developing a standardized approach. Developing training programs and guidelines will ensure that all nurses are proficient in performing auricular massage, thereby enhancing the overall pain management strategy for premature neonates.

## 6. Limitations

The results of the current study demonstrate that auricular massage is effective in alleviating pain caused by naso-oral suction in premature neonates in the NICU. However, it is important to note that the auricular massage protocol used in the study is not standardized due to the absence of established guidelines. Additionally, the study sample primarily consisted of moderate and late premature infants, who weighed more than 1.5 kg and were medically stable. These inclusion criteria were set to ensure a sample of more developed neonates capable of tolerating such stimuli. Consequently, the generalization of the current findings to less mature infants with significant medical challenges is problematic.

Future research should focus on very and extremely premature neonates, who typically face more health challenges and are exposed to more medical procedures that induce painful stimuli. Moreover, the current study was conducted in a single hospital, and we recommend that future studies be carried out in multiple centers to enhance the robustness of the findings. Longitudinal studies would also be beneficial to understand the long-term impact of auricular massage on premature infants' health and developmental outcomes. Finally, the mechanism of pain relief through auricular massage has not been established, which presents opportunities for further investigation.

## 7. Conclusion

In conclusion, auricular massage has been demonstrated to effectively alleviate pain caused by naso-oral suction in premature neonates in the NICU. This nonpharmacological intervention, which requires no equipment and has no adverse effects, offers a promising approach to pain management during a critical period of brain development. The study's rigorous design, including the use of a crossover method and standardized procedures, supports the validity of the findings. Results showed that moderate and late premature neonates can benefit from a 3-min session of auricular massage, as evidenced by significant reductions in neonatal pain scores measured by the PIPP-R scale. However, further research is needed to establish standardized protocols, explore the mechanism of pain relief, and assess the long-term impact on very and extremely premature neonates. Implementing auricular massage in clinical practice could enhance neonatal care by reducing reliance on pharmaceutical methods and fostering greater parental involvement.

## Figures and Tables

**Table 1:** Demographic characteristics of the study sample.

**Variable**	**Frequency/mean (SD)**	**Percent**
Gender		
Male	35	58.3%
Female	25	41.7%
Gestational age (weeks)	M = 34.3 (SD = 1.78)	
Ranges 31‐36 + 6 days		
Type of delivery		
Vaginal	13	21.7
Cesarean	47	78.3
Current weight (grams)	M = 1860 g (SD = 530)	
Apgar scores at 5 min		
7	11	18.3%
8	37	61.7%
9	12	20%
Multiplicity of birth		
Singletons	42	70%
Twins	12	20%
Triplets	6	10%

**Table 2:** Study conditions.

**Characteristics**	**Conditions**		**p** ** value**
	**Condition I**	**Condition II**	
	**First massage then no massage (** **N** = 30**)**	**First no massage then massage (** **N** = 30**)**	
Gender			0.43^§^
Male	16 (53.3%)	19 (63.3%)	
Female	14 (46.7%)	11 (36.7%)	
Gestational age	34.1 (1.87)	34.5 (1.70)	0.41⁣^∗^
Body weight	1794.33 (505.39)	1921.33 (549.42)	0.35⁣^∗^

^§^Significance level obtained by chi-square test.

⁣^∗^Significance level obtained by independent *t*-test.

**Table 3:** Premature infant's overall pain assessments scores across study periods for the two conditions.

**Time for pain assessments**	**Mean of pain level (SD)**		**t** ** -value (df)**	**p** ** value**
	**Condition I ** ^**a**^ ** (** **n** = 30**)**	**Condition II ** ^**b**^ ** (** **n** = 30**)**		
After massage	3.77 (2.92)	3.50 (1.68)	0.43 (58)	0.67
After no massage	10.47 (2.34)	10.00 (2.47)	0.75 (58)	0.46

^a^Neonates initially received auricular massage after suctioning, but after 2 days, they did not receive auricular massage following suctioning.

^b^Neonates initially did not receive auricular massage following suctioning, but after 2 days, they received auricular massage following suctioning.

**Table 4:** The frequencies of neonatal pain levels after suctioning during periods of auricular massage and without auricular massage for both study conditions.

**Pain level**	**Absence of auricular massage, ** **N** ** (%)**	**Auricular massage, ** **N** ** (%)**	**p** ** value chi-square statistics**
			*X* ^2^ = 74.58
Mild	5 (8.3%)	52 (86.7%)	*p* < 0001
Moderate	45 (75%)	8 (13.3%)	
Severe	10 (16.7%)	0 (0%)	

**Table 5:** PIPP-R subscale comparison during auricular massage versus no massage (*N* = 60).

**Indicator**	**Median (25%–75% interquartile range)**		**p** ** value** ^**a**^
	**Auricular massage**	**No auricular massage**	
Physiological factors			
Changes heart rate (beat/min)	0.0 (0.0–0.0)	1.0 (1.0–1.0)	< 0.0001
Reduction in oxygen saturation	0.0 (0.0–0.0)	3.0 (1.25–3.0)	< 0.0001
Behavioral indicators			
Brow bulge	1.0 (1.0–1.0)	2.0 (1.0–2.0)	< 0.0001
Eye squeeze	0.5 (0.0–1.0)	2.0 (1.0–2.0)	< 0.0001
Nasolabial furrow	0.0 (0.0–0.0)	1.0 (1.0–2.0)	< 0.0001
Contextual factors			
Gestational age	1.0 (0.0–1.0)		
Baseline behavioral state	1.0 (1.0–1.0)	2.0 (1.0–2.0)	< 0.0001

*Note:* PIPP-R grading for brow bulge, eye squeeze, and nasolabial furrow: none = 0; minimal = 1; moderate = 2; maximal = 3. Grading for baseline behavioral state: active and awake = 0; quiet and awake = 1; active and asleep = 2; quiet and asleep = 3. Grading for gestational age: >36 weeks = 0; 32‐35 weeks + 6 days = 1; 28‐31 weeks + 6 days = 2; <28 weeks = 3.

^a^Wilcoxon's signed-rank tests.

## Data Availability

The data used to support the findings of this study are available from the corresponding author upon request.

## References

[1] World Health Organization (2023). Preterm Birth. https://www.who.int/news-room/fact-sheets/detail/preterm-birth#:%7E:text=Preterm%2520is%2520defined%2520as%2520babies,to%2520less%2520than%252032%2520weeks.

[2] World Health Organization (WHO) (2023). The Global Health Observatory: Explore a World of Health Data. https://www.who.int/news-room/fact-sheets/detail/preterm-birth.

[3] Balest A. L. (2021). Premature Newborn - Children's Health Issues. MSD Manual Consumer Version. https://www.msdmanuals.com/home/children-s-health-issues/general-problems-in-newborns/premature-newborn.

[4] Cruz M. D., Fernandes A. M., Oliveira C. R. (2016). Epidemiology of Painful Procedures Performed in Neonates: A Systematic Review of Observational Studies. *European Journal of Pain*.

[5] Hatfield L. A., Murphy N., Karp K., Polomano R. C. (2019). A Systematic Review of Behavioral and Environmental Interventions for Procedural Pain Management in Preterm Infants. *Journal of Pediatric Nursing*.

[6] Bonutti D. P., Daré M. F., Castral T. C., Leite A. M., Vici-Maia J. A., Scochi C. G. S. (2017). Dimensioning of Painful Procedures and Interventions for Acute Pain Relief in Premature Infants. *Revista Latino-Americana de Enfermagem*.

[7] Luo F., Zhu H., Mei L. (2023). Evaluation of Procedural Pain for Neonates in a Neonatal Intensive Care Unit: A Single-Centre Study. *BMJ Paediatrics Open*.

[8] Rupawala M., Bucsea O., Laudiano-Dray M. P. (2023). A Developmental Shift in Habituation to Pain in Human Neonates. *Current Biology*.

[9] Hill S., Engle S., Jorgensen J., Kralik A., Whitman K. (2005). Effects of Facilitated Tucking During Routine Care of Infants Born Preterm. *Pediatric Physical Therapy*.

[10] Menon G., McIntosh N. (2008). How Should We Manage Pain in Ventilated Neonates?. *Neonatology*.

[11] Williams M. D., Lascelles B. D. X. (2020). Early Neonatal Pain—A Review of Clinical and Experimental Implications on Painful Conditions Later in Life. *Frontiers in Pediatrics*.

[12] Donia A. E. S., Tolba O. A. (2016). Effect of Early Procedural Pain Experience on Subsequent Pain Responses Among Premature Infants. *Egyptian Pediatric Association Gazette*.

[13] Cook K. M., de Asis-Cruz J., Kim J. H. (2023). Experience of Early-Life Pain in Premature Infants Is Associated With Atypical Cerebellar Development and Later Neurodevelopmental Deficits. *BMC Medicine*.

[14] Cong X., Wu J., Vittner D. (2017). The Impact of Cumulative Pain/Stress on Neurobehavioral Development of Preterm Infants in the NICU. *Early Human Development*.

[15] Selvanathan T., Ufkes S., Guo T. (2024). Pain Exposure and Brain Connectivity in Preterm Infants. *JAMA Network Open*.

[16] Anand K. J. S. (2000). Pain, Plasticity, and Premature Birth: A Prescription for Permanent Suffering?. *Nature Medicine*.

[17] Alorfi N. M. (2023). Pharmacological Methods of Pain Management: Narrative Review of Medication Used. *International Journal of General Medicine*.

[18] Hall R. W., Anand K. J. (2014). Pain Management in Newborns. *Clinics in Perinatology*.

[19] Mangat A. K., Oei J. L., Chen K., Quah-Smith I., Schmölzer G. M. (2018). A Review of Non-Pharmacological Treatments for Pain Management in Newborn Infants. *Children*.

[20] Bucsea O., Riddell R. P. (2019). Non-pharmacological pain management in the neonatal intensive care unit: Managing neonatal pain without drugs. *Seminars in Fetal and Neonatal Medicine*.

[21] Chen K. L., Lindrea K. B., Quah-Smith I. (2017). Magnetic Noninvasive Acupuncture for Infant Comfort (MAGNIFIC) – a Single-Blinded randomised Controlled Pilot Trial. *Acta Paediatrica*.

[22] Gan K. M., Oei J. L., Quah-Smith I. (2020). Magnetic Non-Invasive Auricular Acupuncture During Eye-Exam for Retinopathy of Prematurity in Preterm Infants: A Multicentre Randomized Controlled Trial. *Frontiers in Pediatrics*.

[23] Narciso L. M., Beleza L. O., Imoto A. M. (2022). The Effectiveness of Kangaroo Mother Care in Hospitalization Period of Preterm and Low Birth Weight Infants: Systematic Review and Meta-Analysis. *Journal de Pediatria*.

[24] Niemi A. K. (2017). Review of Randomized Controlled Trials of Massage in Preterm Infants. *Children*.

[25] Özkan T. K., Küçükkelepçe D. Ş., Özkan S. A. (2019). The Effects of Acupressure and Foot Massage on Pain During Heel Lancing in Neonates: A Randomized Controlled Trial. *Complementary Therapies in Medicine*.

[26] Vu-Ngoc H., Uyen N. C. M., Thinh O. P. (2020). Analgesic Effect of Non-Nutritive Sucking in Term Neonates: A Randomized Controlled Trial. *Pediatrics & Neonatology*.

[27] Yavaş S., Bülbül T., Topcu Gavas H. (2021). The Effect on Pain Level and Comfort of Foot Massages Given by Mothers to Newborns Before Heel Lancing: Double-Blind Randomized Controlled Study. *Japan Journal of Nursing Science*.

[28] Alinejad-Naeini M., Mohagheghi P., Peyrovi H., Mehran A. (2014). The Effect of Facilitated Tucking During Endotracheal Suctioning on Procedural Pain in Preterm Neonates: A Randomized Controlled Crossover Study. *Global Journal of Health Science*.

[29] Bagheri F., Vashani H. B., Baskabadi H. A., Tabriz E. R. (2020). An Investigation of the Effects of Massage Therapy on Pain Caused by Umbilical Vein Catheter Insertion in Premature Neonates: A Clinical Trial. *Pakistan Journal of Medical & Health Sciences*.

[30] Dur Ş., Çağlar S., Yıldız N. U., Doğan P., Varal İ. G. (2020). The Effect of Yakson and Gentle Human Touch Methods on Pain and Physiological Parameters in Preterm Infants During Heel Lancing. *Intensive and Critical Care Nursing*.

[31] Fatollahzade M., Parvizi S., Kashaki M., Haghani H., Alinejad-Naeini M. (2022). The Effect of Gentle Human Touch During Endotracheal Suctioning on Procedural Pain Response in Preterm Infant Admitted to Neonatal Intensive Care Units: A Randomized Controlled Crossover Study. *The Journal of Maternal-Fetal & Neonatal Medicine*.

[32] Gomes Neto M., da Silva Lopes I. A., Araujo A. C. C. L. M., Oliveira L. S., Saquetto M. B. (2020). The Effect of Facilitated Tucking Position During Painful Procedure in Pain Management of Preterm Infants in Neonatal Intensive Care Unit: A Systematic Review and Meta-Analysis. *European Journal of Pediatrics*.

[33] Oliveira N. R. G., Formiga C. K. M. R., Ramos B. A. (2023). Gentle Touch and Sucrose for Pain Relief During Suctioning in Preterm Newborns—A Randomized Clinical Trial. *Children*.

[34] Taplak A. Ş., Bayat M. (2021). Comparison the Effect of Breast Milk Smell, White Noise and Facilitated Tucking Applied to Turkish Preterm Infants During Endotracheal Suctioning on Pain and Physiological Parameters. *Journal of Pediatric Nursing*.

[35] Wirz-Ridolfi A. (2019). The History of Ear Acupuncture and Ear Cartography: Why Precise Mapping of Auricular Points Is Important. *Medical Acupuncture*.

[36] Niemtzow R. C. (2007). Battlefield Acupuncture. *Medical Acupuncture*.

[37] Wang J., Zhang J., Sun D. (2022). Randomized Crossover Study of Auricular Plaster Therapy to Relieve Dental Anxiety in Children. *Frontiers in Psychiatry*.

[38] Yao J., Chen L., Zhang L. (2019). Effect of Auriculotherapy and Intervention Types on Weight Control: A Systematic Review and Meta-Analysis Protocol. *Medicine*.

[39] Auriculotherapy Certification Institute (2025). Auriculotherapy. https://www.auriculotherapy.org/.

[40] Marko T., Dickerson M. L. (2017). *Clinical Handbook of Neonatal Pain Management for Nurses*.

[41] Qiu J., Jiang Y. F., Li F., Tong Q. H., Rong H., Cheng R. (2017). Effect of Combined Music and Touch Intervention on Pain Response and *β*-Endorphin and Cortisol Concentrations in Late Preterm Infants. *BMC Pediatrics*.

[42] Zayed D. A., Fathalla A. A. (2022). Crossover Study: Effect of Facilitated Tucking Position on Preterm Infants Pain and Suction Duration During Endotracheal Suctioning. *Egyptian Journal of Health Care*.

[43] Chik Y. M., Ip W. Y., Choi K. C. (2017). The Effect of Upper Limb Massage on Infants’ Venipuncture Pain. *Pain Management Nursing*.

[44] Jain S., Kumar P., McMillan D. D. (2006). Prior Leg Massage Decreases Pain Responses to Heel Stick in Preterm Babies. *Journal of Paediatrics & Child Health*.

[45] Stevens B. J., Gibbins S., Yamada J. (2014). The Premature Infant Pain Profile-Revised (PIPP-R). *The Clinical Journal of Pain*.

[46] Taplak A. Ş., Bayat M. (2019). Psychometric Testing of the Turkish Version of the Premature Infant Pain Profile Revised-PIPP-R. *Journal of Pediatric Nursing*.

[47] Nurbayanti S. (2021). The Effects of Breast Feeding and Massage on Neonatus Pain During Intravenous Blood Sampling Procedures. *Journal of Neonatal Nursing*.

[48] Roshanray A., Rayyani M., Dehghan M., Faghih A. (2020). Comparative Effect of Mother's Hug and Massage on Neonatal Pain Behaviors Caused by Blood Sampling: A Randomized Clinical Trial. *Journal of Tropical Pediatrics*.

[49] Weatherford C. (2022). *The International Association of Infant Massage Programme and Parent-Infant Relationship Outcomes: A Systematic Review [M.S thesis]*.

[50] Cha H. S., Park H. (2020). Effects of Auricular Acupressure on Korean Children Who Are Obese. *Journal of Pediatric Nursing*.

[51] Gao H., Zhang L., Liu J. (2020). Auricular Acupressure for Myopia in Children and Adolescents: A Systematic Review. *Complementary Therapies in Clinical Practice*.

[52] Graff D. M., McDonald M. J. (2018). Auricular Acupuncture for the Treatment of Pediatric Migraines in the Emergency Department. *Pediatric Emergency Care*.

[53] Jackson H. J., Lopez C., Miller S., Englehardt B. (2021). Feasibility of Auricular Acupressure as an Adjunct Treatment for Neonatal Opioid Withdrawal Syndrome (NOWS). *Substance Abuse*.

[54] Yeh C. H., Chien L. C., Chiang Y. C., Lin S. W., Huang C. K., Ren D. (2012). Reduction in Nausea and Vomiting in Children Undergoing Cancer Chemotherapy by Either Appropriate or Sham Auricular Acupuncture Points With Standard Care. *The Journal of Alternative and Complementary Medicine*.

[55] Johnston C. C., Filion F., Campbell-Yeo M. (2008). Kangaroo Mother Care Diminishes Pain From Heel Lance in Very Preterm Neonates: A Crossover Trial. *BMC Pediatrics*.

[56] Choi S., Kim B. (2024). Effect of Auriculotherapy on Stress: A Systematic Review and Meta-Analysis. *Journal of Holistic Nursing*.

[57] Liu M., Tong Y., Chai L. (2021). Effects of Auricular Point Acupressure on Pain Relief: A Systematic Review. *Pain Management Nursing*.

[58] El-sayd H. M., El-Mashad G. M., Mohamed M. Z. E., Abouzouna Z. S. (2023). Comparison of Massage and Prone Position on Heart Rate and Blood Oxygen Saturation Level in Preterm Neonates Hospitalized in Neonatal Intensive Care Units. *Egypt Pediatric Association Gaz*.

[59] Zhang Y., Duan C., Cheng L., Li H. (2023). Effects of Massage Therapy on Preterm Infants and Their Mothers: A Systematic Review and Meta-Analysis of Randomized Controlled Trials. *Frontiers in Pediatrics*.

[60] Fagard J., Esseily R., Jacquey L., O’Regan K., Somogyi E. (2018). Fetal Origin of Sensorimotor Behavior. *Frontiers in Neurorobotics*.

[61] Geary O., Grealish A., Bright A. M. (2023). The Effectiveness of Mother-Led Infant Massage on Symptoms of Maternal Postnatal Depression: A Systematic Review. *PLoS One*.

